# Time to antibiotic therapy and outcome in bacterial meningitis: a Danish population-based cohort study

**DOI:** 10.1186/s12879-016-1711-z

**Published:** 2016-08-09

**Authors:** Jacob Bodilsen, Michael Dalager-Pedersen, Henrik Carl Schønheyder, Henrik Nielsen

**Affiliations:** 1 Department of Infectious Diseases, Aalborg University Hospital, Mølleparkvej 4, 9100 Aalborg, Denmark; 2 Department of Medicine, Vendsyssel Hospital, Hjørring, Denmark; 3 Department of Clinical Microbiology, Aalborg University Hospital, Aalborg, Denmark; 4 Department of Clinical Medicine, Aalborg University, Aalborg, Denmark

**Keywords:** Bacterial meningitis, Antibiotic therapy, Outcome

## Abstract

**Background:**

Community-acquired bacterial meningitis (CABM) is a life-threatening disease and timing of antibiotic therapy remains crucial. We aimed to analyse the impact of antibiotic timing on the outcome of CABM in a contemporary cohort.

**Methods:**

We conducted a population-based cohort study based on chart reviews of all adult cases (>16 years of age) of CABM in North Denmark from 1998 to 2014 excluding patients given pre-hospital parenteral antibiotics. We used modified Poisson regression analyses to compute the adjusted risk ratio (adj. RR) with 95 % confidence intervals (CIs) for in-hospital mortality and unfavourable outcome at discharge by time after arrival to hospital to adequate antibiotic therapy.

**Results:**

We identified 195 adults with CABM of whom 173 patients were eligible for further analyses. The median door-to-antibiotic time was 2.0 h (interquartile range (IQR) 1.0–5.5). We observed increased adjusted risk ratios for in-hospital mortality of 1.6 (95 % CI 0.8–3.2) and an unfavourable outcome at discharge of 1.5 (95 % CI 1.0–2.2, *p* = 0.03) when treatment delays exceeded 6 h versus treatment within 2 h of admission. These findings corresponded to adjusted risk ratios of in-hospital mortality of 1.1 per hour of delay (95 % CI 0.8–1.5) and an unfavourable outcome at discharge of 1.1 per hour of delay (95 % CI 1.0–1.3) within the first 6 h of admission. Some patients (31 %) were diagnosed after admission and had more delays in antibiotic therapy and correspondingly increased in-hospital mortality (30 vs 14 %, *p* = 0.01) and unfavourable outcome (62 vs 37 %, *p* = 0.002).

**Conclusions:**

Delay in antibiotic therapy was associated with unfavourable outcome at discharge.

**Electronic supplementary material:**

The online version of this article (doi:10.1186/s12879-016-1711-z) contains supplementary material, which is available to authorized users.

## Background

Community-acquired bacterial meningitis (CABM) is a devastating disease with substantial morbidity and mortality despite treatment with modern antibiotics, advanced intensive care and adjuvant dexamethasone treatment [1, 2]. Thus far, attempts to further improve outcome, including supplemental treatment with glycerol and induced hypothermia, have failed [3–6].

Therefore, timing of initiation of antibiotic therapy in CABM and potential consequences of delays remains a topic of major interest. Some studies addressing door-to-antibiotic time have had inconclusive results, but most reports suggest more unfavourable outcomes with delayed antibiotic administration [7–12]. However, the two most cited studies were based on study populations from 1990-2002 and 2002–2004, respectively, and they consisted of relatively small cohorts in the era prior to standard use of adjuvant dexamethasone therapy [11, 12]. To our understanding, these studies did not account for whether any patients were treated with parenteral antibiotics before admission, which has been associated with a high mortality elsewhere [13, 14]. Moreover, these studies did not specify if patients were diagnosed at admission to the emergency department or later. This could confound analyses of delays in antibiotic therapy and outcome as these patients may have increased mortality and morbidity due to other reasons, e.g. predominance of different bacterial aetiologies, impaired immunological response, comorbidity etc. Therefore, timing of antibiotic therapy and outcome needs further clarification in a contemporary cohort.

We conducted a retrospective study of adult patients with CABM in North Denmark over the last 17 years with the aim of examining time to antibiotic therapy and outcome.

## Methods

### Setting

We carried out a population-based cohort study in patients with CABM from 1 January 1998 to 31 December 2014 in North Denmark Region. The catchment population was approximately 500,000 in 1998 and approximately 580,000 in 2014 [15]. In Denmark, primary health-care and hospital care are tax-paid and free of charge. A unique personal identification number is provided to all residents at birth or immigration and is used for all health-care contacts.

Owing to health administrative reforms, the number of hospitals providing acute care for patients decreased from nine in 1998 to five in 2014. The Department of Clinical Microbiology at Aalborg University Hospital was the sole provider of microbiological diagnostics for every hospital in the region throughout the entire study period. This department also kept records of specimens sent for supplementary analyses at Statens Serum Institut, Copenhagen.

Since 1998 local guidelines recommended intravenous penicillin G (supplemented with gentamicin in patients aged above 40 years) as empiric treatment for CABM and since 2003 adjuvant dexamethasone was added. In 2009, the advised antibiotic regimen was replaced by the combination of penicillin G and cefotaxime to all adults according to national Danish guidelines. Throughout the study period, cerebrospinal fluid isolates of *Streptococcus pneumoniae* and *Neisseria meningitidis* in Denmark have retained wildtype sensitivity to penicillin (94.7 and >99 % of isolates, respectively), and high-level resistant isolates of pneumococci have been rare [16, 17].

### Study population

The laboratory information system (ADBakt, Autonik, Sweden) at the Department of Clinical Microbiology, Aalborg University Hospital, was used to identify cases of CABM in North Denmark Region. All patients aged over 16 years were included if they had a clinical presentation strongly suggestive of CABM, including signs such as neck stiffness, petechiae, headache, fever, confusion, or impaired level of consciousness, and at least one of the following:

1. Positive CSF culture
2. Positive blood culture and more than >10 leukocytes × 10^6^/L in the CSF [18].
3. Presence of bacteria in Gram stain of CSF
4. Non-culture detection of bacteria in CSF by either bacterial antigen test or 16S rRNA gene amplification.

If a patient fulfilled multiple criteria, only the strongest criterion was noted (1>2>3>4). Patients with concomitant infections, e.g. endocarditis or spondylodiscitis, were included if their initial main presentation was characteristic of meningitis and they fulfilled one of the above inclusion criteria.

Exclusion criteria were: cerebral abscess, hospital-acquired bacterial meningitis as defined by the Centers for Disease Control [19], an implanted neurosurgical device, or cases where an exact time of antibiotic treatment or the clinical records could not be retrieved.

### Data sources

The main author reviewed all patient records. Time of arrival at hospital was retrieved from ambulance charts or, secondarily, from registrations made by secretaries or nurses at admission to the Emergency Department. Detailed information on patient history, pre-hospital antibiotic treatment, clinical findings, preliminary diagnoses at admission and outcome were obtained from the ambulance, nurses’ and doctors’ charts. When available, letters of referral from general practitioners and ambulance records were checked for remarks indicating suspicion of bacterial meningitis. Electronic records systems were used to obtain timing and results of diagnostic tests, i.e. blood tests (Labka I and Labka II, CSC, USA), microbiological samples (ADBakt) and cranial imaging (Easyviz, Karos Health, Denmark). For patients admitted to an intensive care unit (ICU), observation charts of vital signs and medical treatment were examined.

Sources for time of antibiotic therapy included doctors’, nurses’ and ICU charts, emergency triage notes, and manual medication administration records. These also included the electronic medication administration systems Theriak (TM Software, Iceland) from 2004 to 2012 and Opus (CSC, USA) from 2012 onwards.

### Time to antibiotic therapy

To obtain the time to antibiotic therapy, we prioritised nurses’ signatures for antibiotic administration on triage forms or on nurses’ charts or in medication administration systems – paper and electronic – unless an exact time of treatment was specified elsewhere, e.g. in the doctors’ charts. Time to antibiotic therapy was calculated as time from arrival at hospital to administration of first dose of antibiotic therapy providing coverage for the specific bacterial aetiology and antibiogram in the given patient in dosages recommended for treating bacterial meningitis.

### Patient data

The criterion ‘diagnosed with meningitis at referral to hospital’ was defined as when the referral letter from the general practitioner or the ambulance records mentioned suspicion of bacterial meningitis. The criterion ‘diagnosed with bacterial meningitis at admission’ denotes patients listed above and/or where meningitis was mentioned in the admission records. This also included cases that had a lumbar puncture performed or planned at admission and patients started on antibiotic therapy for bacterial meningitis. All other cases were regarded as diagnosed later.

Baseline data were collected for the day of admission in all patients. Symptoms or clinical findings were categorised as not present if they were not mentioned in the medical records. Adjuvant dexamethasone was registered if it was administered (10 mg four times daily for 4 days) within 1 h of intravenous antibiotic treatment. We categorised disseminated intravascular coagulation (DIC) at admission as thrombocytopenia (platelet count below 150 × 10^9^/L) and an activated plasmatic coagulation profile and/or petechiae or ecchymoses with systemic complications. An exact Glasgow Coma Scale (GCS) score at admission was reported in about two-thirds of the records, and if not reported, a categorical classification was made based on observations in the patients’ records (GCS < 9, GCS 9–12, GCS > 12). Impaired mental status was noted if the patient had any level of reduced consciousness not explained by previous comorbidity. The duration of hospitalisation was registered from the day of admission to the day of discharge or transfer to a rehabilitation unit.

### Outcome

Outcome was graded according to the Glasgow Outcome Scale (GOS) at discharge [20]. The scores and their corresponding conditions are as follows: 1, death; 2, a vegetative state (unable to interact with the environment); 3, severe sequelae and dependency upon others in daily life; 4, moderate sequelae but retainment of the capability of independent living; and 5, no – or only minor – sequelae. A score of 5 was considered favourable and 1–4 as unfavourable.

### Statistical analysis

Categorical data were analysed using Fisher’s exact test or the *χ*^2^ test, and continuous data were analysed using the Mann–Whitney *U*-test. A two-tailed *p*-value <0.05 was considered significant. Because odds ratio are difficult to interpret when events are common, we computed the risk and risk ratio for the primary outcomes in-hospital mortality and unfavourable outcome at discharge by time to adequate antibiotic therapy [21]. To compute the adjusted risk ratios (adj. RRs) with 95 % confidence intervals (95 % CIs), we used modified Poisson regression analyses [22]. Due to the number of events we could only adjust for age >65 years, GCS score at admission (GCS < 13), and presence of hypotension (systolic blood pressure <90 mmHg) in analyses of in-hospital mortality (33 events) with further adjustment for bacterial aetiology (*S. pneumoniae* yes/no) and adjunctive dexamethasone treatment in analyses of unfavourable outcome at discharge (77 events). In regression analyses, we omitted patients who had been treated with parenteral beta-lactams prior to admission.

We used Stata 11.2 (StataCorp., College Station, TX) for all analyses.

## Results

We identified 195 adult cases of CABM during the study period. In six cases we could not identify an accurate time of the first adequate dose of antibiotics and in two cases the patient records could not be retrieved. Another patient had died at home and was diagnosed by a forensic autopsy. Prior to admission 13 patients had been treated by the referring general practitioners with intramuscular benzylpenicillin 3.0 g on suspicion of bacterial meningitis, seven (53 %) of whom died (Additional file 1: Table S1). Therefore, 22 patients were excluded, with 173 patients remaining for further analysis of time to antibiotic therapy.

**Fig. 1:**
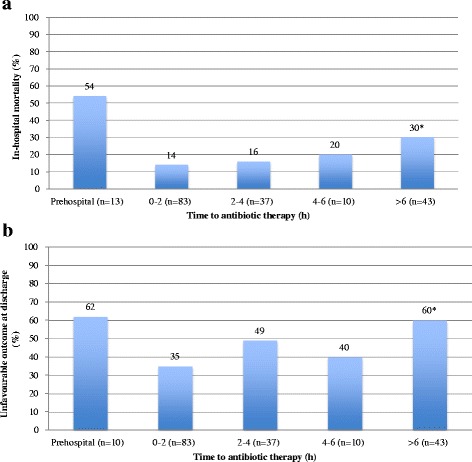
**a** Time to antibiotic therapy and in-hospital mortality in community-acquired bacterial meningitis. *P-value <0.05 compared with patients treated 0–2 h from admission. **b** Time to antibiotic therapy and unfavourable outcome at discharge in community-acquired bacterial meningitis. *P-value <0.05 compared with patients treated 0–2 h from admission

Baseline characteristics of the patients are presented in Table 1 and the bacterial aetiologies are shown in Additional file 2: Table S2. Examining all 173 patients, the median time to antibiotic therapy was 2.0 h (IQR 1.0–5.5). We observed that increased time to antibiotic therapy was associated with in-hospital mortality (Fig. 1a, *p* = 0.04) and unfavourable outcome at discharge (Fig. 1b, *p* = 0.01), reaching statistical significance when treatment delays exceeded 6 h compared with patients treated within 2 h of admission. Adjusted risk ratios of in-hospital mortality and an unfavourable outcome at discharge were 1.6 (95 % CI 0.8–3.2) and 1.5 (95 % CI 1.0–2.2, *p* = 0.03), respectively, in patients treated more than 6 h after admission, using patients treated within 2 h as reference (Table 2). These findings corresponded to an adjusted risk ratio of in-hospital mortality of 1.1 per hour of delay (95 % CI 0.8–1.5) and an unfavourable outcome at discharge of 1.1 per hour of delay (95 % CI 1.0–1.3) during the first 6 h of admission.

**Table 1:** Baseline characteristics at admission of patients with community-acquired bacterial meningitis excluding patients treated with pre-hospital parenteral antibiotics

Patient characteristic at admission (*n* = 173)	Total cohort
Age (years)	58 (45–70)
Male	84 (49)
Comorbidity^a^	62/173 (36)
Diagnosis of meningitis suspected at referral to hospital	35/173 (20)
Diagnosed with meningitis at admission to hospital	120/173 (69)
Duration of symptoms (days) (*n* = 164)	2 (2–5)
Headache	96/119 (81)
Nausea/vomiting	87/102 (85)
Petechiae/echimoses	40/135 (30)
Neck stiffness	113/160 (71)
Focal neurological deficit^b^	29/130 (22)
New-onset seizures	17/173 (10)
Meningitis triad (neck stiffness, fever, impaired mental status)	78/173 (45)
Temperature (°C) (*n* = 169)	38.9 (38.0–39.9)
Blood pressure, systolic (mmHg) (*n* = 157)	138 (119–159)
Pulse rate (bpm) (*n* = 156)	99 (87–116)
Glasgow Coma Score	
12–15	82 (47)
9–12	61 (35)
< 9	30 (17)
C-reactive protein (mg/L) (*n* = 170)	211 (110–298)
Blood leukocytes (10^9^/L) (*n* = 171)	17 (11–24)
Disseminated intravascular coagulopathy	31/173 (18)
CSF	
Leukocytes (10^6^/L) (*n* = 164)	1925 (204–6081)
Erythrocytes (10^6^/L) (*n* = 164)	145 (18–593)
CSF-glucose (mmol/L) (*n* = 163)	0.9 (0.1–3.1)
CSF/blood glucose ratio (*n* = 163)	0.1 (0.01–0.4)
CSF protein (g/L) (*n* = 166)	3.7 (1.9–7.9)
Aetiology	
*S. pneumoniae*	96/173 (55)
*N. meningitidis*	36/173 (21)
Other	41/173 (24)
Antibiotics for sepsis given before meningitis was diagnosed	11/173 (6)
Time to antibiotic therapy for meningitis (h, *n* = 173)	2.0 (1.0–5.5)
Dexamethasone treatment^c^	55/116 (47)
Cranial imaging before lumbar puncture	60/173 (35)
Cranial imaging during hospitalisation	134/173 (77)
Intensive care unit admission	104/173 (60)
Unfavourable outcome (GOS 1–4)	77/173 (45)
Mortality	33/173 (19)

Binary variables are listed as n/N (%) and continuous variables as medians (IQR). ^a^Alcoholism, asplenia, cancer, cirrhosis, congenital or acquired immunodeficiency including HIV, diabetes mellitus, heart failure (ejection fraction <40 %) and renal impairment (serum creatinine >130 μmol/L). ^b^Paraesthesia, motor or cranial nerve paresis. ^c^Patients included after implementation of adjunctive dexamethasone treatment in 2002

**Table 2:** Time to adequate antibiotic therapy and risk ratios for in-hospital mortality and unfavourable outcome at discharge among patients with community-acquired bacterial meningitis

Adequate antibiotic therapy initiated	Risk ratio for in-hospital mortality (95 % CI)		Risk ratio for unfavourable outcome (95 % CI)	
	Crude	Adjusted^a^	Crude	Adjusted^b^
0–2 h after admission	Ref.	Ref.	Ref.	Ref.
2–4 h after admission	1.1 (0.5–2.8)	1.2 (0.5–2.7)	1.4 (0.9–2.2)	1.5 (0.9–2.2)
4–6 h after admission	1.4 (0.4–5.3)	1.4 (0.5–3.6)	1.1 (0.5–2.6)	1.1 (0.6–2.2)
>6 h after admission	2.1 (1.0–4.2)*	1.6 (0.8–3.2)	1.7 (1.2–2.5)*	1.5 (1.0–2.2)*

*p < 0.05. CI indicates confidence interval. ^a^Adjusted for age >65 years, Glasgow Coma Score <13 and arterial systolic hypotension (<90 mmHg) at admission. ^b^Adjusted for age >65 years, Glasgow Coma Score <13, arterial systolic hypotension (<90 mmHg), bacterial aetiology (*S. pneumoniae* yes/no) and adjunctive dexamethasone treatment (yes/no)

Comparing patients diagnosed immediately at admission with patients diagnosed after admission, we found a median time to antibiotic therapy of 1.3 h (interquartile range (IQR) 0.8–2.7) vs 8.5 h (IQR 3.9–24.5) (*p* < 0.001), and correspondingly in-hospital mortality of 14 vs. 30 % (*p* = 0.01) and an unfavourable outcome at discharge of 37 vs. 62 % (*p* = 0.002).

Similarly, patients presenting without the meningitis triad (fever, neck stiffness, altered mental status) had longer door-to-antibiotic time (median 3.1 vs. 1.3 h, *p* < 0.001), but no substantial differences in in-hospital mortality (16 vs. 23 %, *p* = 0.3) and unfavourable outcome (41 vs. 49 %, *p* = 0.4).

Cranial imaging before lumbar puncture was more frequent in patients diagnosed after admission (58 vs. 24 %, *p* < 0.001) and was associated with a substantial treatment delay (median door-to-antibiotics 3.1 vs. 1.5 h, *p* = 0.01). A trend towards increased in-hospital mortality (26 vs 16 %, *p* = 0.1) and unfavourable outcome at discharge (51 vs. 41 %, *p* = 0.3) was also found.

Antibiotic delay of more than 2 h after admission in patients with a pre-hospital diagnosis of meningitis (*n* = 35) showed a trend towards increased mortality (RR 1.4; 95 % CI 0.1–14.6) and unfavourable outcome at discharge (RR 1.2; 95 % CI 0.4–3.9). Further details on treatment and outcome according to time of day of admission, bacterial aetiology and duration of symptoms are available in Additional file 3: Table S3.

## Discussion

In this contemporary population-based cohort study of CABM, we observed a median time to antibiotic therapy of 2.0 h after admission. We found a 10 % increase of in-hospital mortality and risk for unfavourable outcomes at discharge with each hour of delay for the first 6 h of admission (of note the increase was non-linear for unfavourable outcome). Antibiotic delays of more than 6 h resulted in a 50–60 % relative increase in in-hospital mortality and unfavourable outcome at discharge when compared with patients receiving adequate antibiotic therapy within 2 h of admission, although statistical significance was not reached in adjusted analyses regarding in-hospital mortality. When compared to patients diagnosed with CABM on admission, patients diagnosed after admission had increased time to antibiotics (8.5 vs. 1.3 h, *p* < 0.001) and mortality (30 vs. 14 %, *p* = 0.01).

The median time to adequate antibiotic therapy in our study was faster than that reported by Proulx et al. (3.8 h) [11] but comparable to observations in another Danish study by Køster-Rasmussen et al. (2.0 h) [12]. Proulx et al. observed increased case fatality (OR 8.4, 95 % CI 1.7–40.9) when treatment delays exceeded 6 h after admission, but without any sign of increased mortality with less delay. Our study consistently showed an increased in-hospital mortality and unfavourable outcome with step-wise delays in time to antibiotic therapy underscoring the biological rationale of the association. Similar to our study, Køster-Rasmussen et al. also found increased unfavourable outcomes with treatment delays (OR 1.1 per hour of delay) in adjusted analyses, but without any report on effect on mortality. Both studies were based on less contemporary cohorts, included fewer adult cases (118 and 125 cases, respectively), and did not specify if patients were given pre-hospital parenteral antibiotics. Furthermore, these studies did not account for time of diagnosis of CABM, which had a substantial effect on antibiotic timing and outcome in our study.

A recently published prospective Swedish observational study with 609 adult cases from 2005 to 2012 also found a median time to antibiotic treatment of 2 h after admission and an improved survival after an amendment of national guidelines limiting the indications for cranial imaging before lumbar puncture [23]. However, information was incomplete regarding time of diagnosis and disease severity (e.g. occurrence of seizures, signs of systemic compromise, and CSF leukocyte count). The study also included a few patients without a proven diagnosis of meningitis, and the completeness of patient inclusion varied during the study period (increasing from 60 to 80 %) [23].

Our study has both strengths and limitations when examining time to antibiotic therapy and outcome of CABM. The population-based design and the inclusion of uniquely identifiable adults limited selection bias, and the validity of the diagnoses was ensured by rigorous inclusion criteria comprising clinical description, CSF findings and microbiological diagnostics. Moreover, we only included cases with an exact time of antibiotic treatment listed in the charts, although we cannot exclude random error (e.g. inaccurate signing off of antibiotic administration by nurses). We also accounted for patients treated with pre-hospital parenteral antibiotics and time of diagnosis.

Several limitations inherent to a retrospective observational study exist, including incomplete data of certain clinical characteristics (e.g. time to antibiotic therapy and GCS score at admission in some patients). We may also have missed a small number of blood-culture negative cases admitted at other departments without a lumbar puncture performed during admission. Differential misclassification in timing of antibiotic therapy may also be present because patients diagnosed immediately at admission more often had an exact point of time documented in their records compared with patients with more uncharacteristic presentations diagnosed later during admission. Moreover, data on time to antibiotic therapy were derived from different data sources without a fixed prioritised order.

Patients diagnosed after admission experienced treatment delay and increased in-hospital mortality and unfavourable outcome at discharge. Patients without typical signs of CABM at admission also had treatment delay, but in this group we found no clear differences in mortality and unfavourable outcome. Still, as the prognosis for these patients remain poor, a high index of suspicion of CABM seems prudent even in patients without all the typical signs of CNS infection.

Duration of disease may be crucially linked to disease severity and thereby outcome, but categorisation of prodromal disease and onset of meningitis is almost impossible in clinical settings, and even more so in retrospective studies. Therefore, we cannot exclude residual confounding although we reported data on disease duration exactly as stated in the medical records. Moreover, we did not see differences in outcome according to duration of symptoms for >24 or <24 h.

## Conclusion

In conclusion, delayed initiation of antibiotic therapy for bacterial meningitis is associated with increased in-hospital mortality and an unfavourable outcome at discharge.

## Abbreviations

CABM, community-acquired bacterial meningitis; CIs, confidence intervals; CSF, cerebrospinal fluid; GCS, Glasgow coma scale; GOS, Glasgow outcome score; ICU, intensive care unit; IQR, interquartile ranges; OR, odds ratios; RR, risk ratios

## Additional files

Additional file 1: Table S1. Selected clinical variables of patients with community-acquired bacterial meningitis treated with pre-hospital parenteral antibiotics. (DOCX 14 kb) Additional file 2: Table S2. Bacterial aetiologies of community-acquired bacterial meningitis in North Denmark Region, 1998–2014. (DOCX 14 kb) Additional file 3: Table S3. Time to antibiotic therapy and outcome according to time of day of admission, duration of symptoms, and bacterial aetiology during the study period. (DOCX 15 kb)
