# Supplementary material for: Time to antibiotic therapy and outcome in bacterial meningitis: a Danish population-based cohort study
Source: BMC Infect Dis. 2016 Aug 9;16:392. doi: 10.1186/s12879-016-1711-z (PMC4977612; doi:10.1186/s12879-016-1711-z)
Supplement: Additional file 1: Table S1. — Selected clinical variables of patients with community-acquired bacterial meningitis treated with pre-hospital parenteral antibiotics. (DOCX 14 kb) [file 12879_2016_1711_MOESM1_ESM.docx]

**Additional file 1**

**Table S1:** Characteristics of 13 patients treated with parenteral benzylpenicillin by the referring doctor on suspicion of bacterial meningitis before admission to hospital.

|  | **Neck stiffness** | **Petechiae** | **In-hospital mortality** |
| --- | --- | --- | --- |
| ***N. meningitidis* (n=8)** | 6/8 | 5/8 | 3/8 |
| ***S. pneumoniae* (n=4)** | 4/4 | 1/4 | 3/4 |
| ***S. anginosus* (n=1)** | 1/1 | 0/1 | 1/1 |
