# Supplementary material for: Time to antibiotic therapy and outcome in bacterial meningitis: a Danish population-based cohort study
Source: BMC Infect Dis. 2016 Aug 9;16:392. doi: 10.1186/s12879-016-1711-z (PMC4977612; doi:10.1186/s12879-016-1711-z)
Supplement: Additional file 2: Table S2. — Bacterial aetiologies of community-acquired bacterial meningitis in North Denmark Region, 1998–2014. (DOCX 14 kb) [file 12879_2016_1711_MOESM2_ESM.docx]

**Additional file 2**

**Table S2:** Bacterial aetiology in community-acquired bacterial meningitis in North Denmark, 1998–2014

| **Bacterial aetiology** | **Total (n)** |
| --- | --- |
| *Streptococcus pneumoniae^a^* | 96 (55) |
| *Neisseria meningitides* | 36 (21) |
| *Staphylocccus aureus* | 9 (5) |
| Haemolytic streptococci | 9 (5) |
| *Listeria monocytogenes* | 8 (5) |
| *Escherichia coli* | 6 (3) |
| *Haemophilus influenza* | 3 (2) |
| Non-haemolytic streptococci | 2 (1) |
| *Enterococcus faecalis* | 2 (1) |
| *Pasteurella multocida* | 1 (1) |
| *Capnocytophaga canimorsus* | 1 (1) |
| Total | 173 |

Of note, 8 of 173 patients presenting with meningitis had accompanying bacterial endocarditis and 4 had spondylodiscitis. In patients admitted from 1998 to 2008 three isolates (two *E. coli* and one *S. aureus*) would not have been treated adequately by the recommended antibiotic regimen at that time (benzylpenicillin + gentamicin). However, two of these isolates (one *E.coli* and one *S. aureus*) were actually treated with relevant high-dose antibiotics for their meningitis within 0.5 and 7.5 hours from admission, respectively (ampicillin and cefuroxime). ^a^One isolate was intermediately resistant and another highly resistant to penicillin.
