# Supplementary material for: Time to antibiotic therapy and outcome in bacterial meningitis: a Danish population-based cohort study
Source: BMC Infect Dis. 2016 Aug 9;16:392. doi: 10.1186/s12879-016-1711-z (PMC4977612; doi:10.1186/s12879-016-1711-z)
Supplement: Additional file 3: Table S3. — Time to antibiotic therapy and outcome according to time of day of admission, duration of symptoms, and bacterial aetiology during the study period. (DOCX 15 kb) [file 12879_2016_1711_MOESM3_ESM.docx]

**Additional file 3**

**Table S3.** Time to antibiotic therapy and outcome according to time of day of

admission, duration of symptoms, and bacterial aetiology during the study period.

|  | **Time to antibiotic therapy (h, IQR)** | **Unfavourable outcome (%)** | **In-hospital mortality (%)** |
| --- | --- | --- | --- |
| **Time of admission^a^** |  |  |  |
| 08:00-15:59 | 2.5 (1.1-7.3) | 41/81 (51) | 19/81 (23) |
| 16:00-23:59 | 1.7 (0.9-5.0) | 23/66 (35) | 9/66 (14) |
| 00:00-07:59 | 2.3 (1.0-3.3) | 13/26 (50) | 5/26 (19) |
| **Duration of symptoms before admission** |  |  |  |
| <24 hours | 2.7 (0.9-5.5) | 12/30 (40) | 7/30 (23) |
| >24 hours | 2.0 (1.0-5.5) | 65/143 (45) | 26/143 (18) |
| **Bacterial aetiology** |  |  |  |
| *S. pneumoniae* | 2.0 (1.1-5.0) | 44/96 (46) | 15/96 (16) |
| *N. meningitidis* | 1.1 (0.6-1.9) | 7/36 (19) | 2/36 (6) |
| Other | 3.9 (1.6-25.1) | 26/41 (45) | 16/41 (39) |

^a^There were no differences in baseline characteristics of the patients admitted at different times of the day, e.g. presence of the meningitis triad (fever, neck stiffness and altered mental status) or not.
